# Characterisation of SARS-CoV-2 Lentiviral Pseudotypes and Correlation between Pseudotype-Based Neutralisation Assays and Live Virus-Based Micro Neutralisation Assays

**DOI:** 10.3390/v12091011

**Published:** 2020-09-10

**Authors:** Inesa Hyseni, Eleonora Molesti, Linda Benincasa, Pietro Piu, Elisa Casa, Nigel J Temperton, Alessandro Manenti, Emanuele Montomoli

**Affiliations:** 1 VisMederi Research s.r.l., 53100 Siena, Italy; hyseni@vismederiresearch.com (I.H.); linda.benincasa@vismederiresearch.com (L.B.); casa@vismederiresearch.com (E.C.); alessandro.manenti@vismederiresearch.com (A.M.); montomoli@unisi.it (E.M.); 2 VisMederi s.r.l., 53100 Siena, Italy; pietro.piu@vismederi.com; 3 Viral Pseudotype Unit, Medway School of Pharmacy, The Universities of Kent and Greenwich at Medway, Chatham ME7 4TB, UK; n.temperton@kent.ac.uk; 4 Department of Molecular and Developmental Medicine, University of Siena, 53100 Siena, Italy

**Keywords:** SARS-CoV-2, wild type virus, lentiviral pseudotypes, biosafety, microneutralisation, serological assays

## Abstract

The recent outbreak of a novel Coronavirus (SARS-CoV-2) and its rapid spread across the continents has generated an urgent need for assays to detect the neutralising activity of human sera or human monoclonal antibodies against SARS-CoV-2 spike protein and to evaluate the serological immunity in humans. Since the accessibility of live virus microneutralisation (MN) assays with SARS-CoV-2 is limited and requires enhanced bio-containment, the approach based on “pseudotyping” can be considered a useful complement to other serological assays. After fully characterising lentiviral pseudotypes bearing the SARS-CoV-2 spike protein, we employed them in pseudotype-based neutralisation assays in order to profile the neutralising activity of human serum samples from an Italian sero-epidemiological study. The results obtained with pseudotype-based neutralisation assays mirrored those obtained when the same panel of sera was tested against the wild type virus, showing an evident convergence of the pseudotype-based neutralisation and MN results. The overall results lead to the conclusion that the pseudotype-based neutralisation assay is a valid alternative to using the wild-type strain, and although this system needs to be optimised and standardised, it can not only complement the classical serological methods, but also allows serological assessments to be made when other methods cannot be employed, especially in a human pandemic context.

## 1. Introduction

In early December 2019, cases of severe pneumonia of unknown aetiology were reported by the China Health Authority. In January 2020, a novel coronavirus was identified as 2019-nCoV (subsequently renamed as SARS-CoV-2).

An initial site of infections was the Huanan seafood market, where live animals are sold. Progressively, human-to-human transmission occurred [1], causing a disease named coronavirus disease 2019 (COVID-19). On 20th July 2020, the World Health Organization (WHO) estimated the global incidence of COVID-19 as 14,348,858 cases and the number of the deaths as 603,691 [2].

SARS-CoV-2 is a member of the *Coronaviridae* family, which comprises two subfamilies of enveloped, positive-stranded RNA viruses. The subfamilies of *Coronavirinae* are classified in four genera: alpha-CoV, beta-CoV, gamma-CoV and delta-CoV [3].

Genome sequence analysis has shown that SARS-CoV-2 belongs to the Betacoronavirus genus, which includes Bat SARS-like coronavirus, Severe Acute Respiratory Syndrome coronavirus (SARS-CoV) and Middle Eastern Respiratory Syndrome coronavirus (MERS-CoV) [4].

The SARS-CoV-2 genome contains four main structural proteins: the spike (S), membrane (M), envelope (E) and nucleocapsid (N) protein [5,6].

The spike (S) protein of coronaviruses, a type I membrane glycoprotein expressed on the viral surface, mediates the attachment of SARS-CoV-2 to the target cells and its subsequently entry. As previously shown in the case of SARS-CoV [7,8], the SARS-CoV-2 S protein engages angiotensin-converting enzyme 2 (ACE2) as its host target receptor. ACE2 is the main host cell receptor of SARS-CoV-2 and plays a crucial role in the entry of the virus into the cells [9].

In addition, viral entry requires S protein priming by cellular proteases, such as the serine protease TMPRSS2, which allows the fusion of viral and cellular membranes to fuse [10,11]. As a result, the spike is cleaved into two subunits: the N-terminal domain, called S1, which is responsible for receptor binding [12,13,14,15], and a C-terminal S2 domain, which is responsible for fusion [14,15].

As the coronavirus S glycoprotein is surface-exposed and mediates entry into host cells, it is the main target of neutralising antibodies (Abs) [16] upon infection, and therefore, the focus of therapeutic strategies and vaccine design.

However, since SARS-CoV-2 displays marked pathogenicity (COVID-19) [17], working with the live virus (LV) implies the need for high biosafety levels laboratories (BSL3). By contrast, the lentiviral pseudotypes system, which has already been successfully adopted in the fight against emerging and re-emerging viruses, constitutes a useful, safe and versatile tool for studies on potential vaccines and therapies. Indeed, the lentiviral pseudotype platform can be efficiently used in conventional biosafety conditions to study cell type susceptibility on the bases of the cell’s expression of ACE2 and protease priming [18,19,20]. Moreover, pseudotypes bearing the spike S-protein of the novel SARS-CoV-2 could prove essential for antibody detection and for the evaluation of neutralisation activity in association with the well-characterised serological methods, such as the Enzyme-linked immunoassay (ELISA) and Micro-Neutralisation assay (MN) [21,22,23].

In the present study, we described the production and characterisation of lentiviral pseudotype particles bearing the SARS-CoV-2 S protein and used these to study S-protein-mediated cell entry. Subsequently, SARS-CoV-2 pseudotypes were also used to measure neutralising antibody responses in serum samples derived from a subset of subjects involved in a sero-epidemiological study in the Tuscany region of Italy during the 2019 outbreak and a panel of negative confirmed sera.

The results were compared with the data obtained when the SARS-CoV-2 live virus was tested in the MN assay.

## 2. Materials and Methods

### 2.1. Cell Line Cultures

HEK 293 T/17 cells (Human embryonic kidney 293 cells) (ATCC–CRL 1573), MDCK cells (Madin-Darby Canine Kidney cells) (ATCC^®^ CCL-34), Vero E6 cells (Epithelial cell line from the kidney of a normal monkey Cercopithecus aethiops) (ATCC–CRL 1586) and Caco2 cells (epithelial cell line from Human Colorectal Adenocarcinoma) (ATCC HTB37) were acquired from the American Type Culture Collection.

Huh-7 cells (Epithelial cell line from Human hepatocellular carcinoma) (ECACC—Code 01042712) and Hep G2 cells (Human Caucasian hepatocyte carcinoma) were provided by the University of Siena, Italy.

Hep G2 cells were cultured in RPMI 1640 (Gibco) supplemented with 2 mM L-Glutamine (Lonza, Milan, Italy), 100 units/mL penicillin-streptomycin (Lonza, Milano, Italy) and 10% foetal bovine serum (FBS) (Euroclone, Pero, Italy).

HEK293 T/17, MDCK and Huh-7 cell lines were cultured in Dulbecco’s Modified Eagle’s Medium (DMEM) High Glucose (Euroclone, Pero, Italy) supplemented with 2 mM L-Glutamine (Lonza, Milan, Italy), 100 units/mL penicillin-streptomycin (Lonza, Milan, Italy) and 10% of FBS (FBS Euroclone, Pero, Italy).

Vero E6 and Caco2 cells were cultured in Eagle’s Minimum Essential Medium (EMEM) (Lonza, Milan, Italy) supplemented with 2 mM L-Glutamine (Lonza, Milan, Italy), 100 units/mL penicillin-streptomycin (Lonza, Milan, Italy) and FBS (Euroclone, Pero, Italy) to a final concentration of 10% for Vero E6 and 20% for Caco2.

All the cell lines used were incubated at 37 °C, 5% CO_2_ in humidified atmosphere and were sub-cultured twice a week until passage 20.

### 2.2. Serum Samples

A total of 65 samples from an Italian sero-epidemiological study, anonymously collected in compliance with Italian ethics law, were provided by the laboratory of Molecular Epidemiology of the University of Siena, Italy. The human sera, derived from a sero-epidemiological study, had previously been tested in an ELISA assay as pre-screening, and positive, borderline and negative ELISA samples were tested in a micro-neutralisation assay, as previously described [24]. This panel of sera was subsequently tested with SARS-CoV-2 pseudotypes in order to compare the neutralisation profiles when they were tested against the live virus and the surrogate virus. As an internal positive control, a panel of samples collected from health care workers (confirmed positive for SARS-CoV-2 by Reverse Real-Time PCR) were kindly provided by Prof. Valentina Bollati, University of Milan.

In addition, three monoclonal antibodies (mAbs) were included in the serological assay: Human IgG1 anti-S1 CR3022 (Native Antigen, Oxford, UK), Human IgG1 Anti-RBD (eEnzyme, Gaithersburg, MD, USA) and Human Anti-IgM SARS-CoV-2 spike S1 CR3022 (Absolute Antibody, 21 Drydock Avenue, 7th Floor Boston, MA 02210, USA (1:100 starting dilutions).

### 2.3. Production, Quantification and Characterisation of Lentiviral Pseudotypes with S Protein from SARS-CoV-2

#### 2.3.1. Plasmids

The full-length S protein (GenBank accession number: YP_009724390.1) was codon-optimised and synthesised (GenScript, Cina), and the S fragment was cloned into the expression vector as described previously [25]. The HIV *gag-pol* plasmid (p8.91) [26], the firefly luciferase-expressing plasmid (PCSFLW) [27], the pCAGGS-TMPRSS2 plasmid and the plasmid encoding for the spike’s human ACE2 receptor were kindly provided by Dr. Nigel Temperton and have previously been described [28,29,30].

As a control, a vesicular stomatitis virus (VSV-G) plasmid was used (pCMV-VSV-G) (Addgene plasmid 8454; http://n2t.net/addgene:8454) [31]. The day before transfection, confluent plates of HEK 293T/17 cells were split 1:4 and seeded into 10 cm^2^ plates in DMEM 10% FBS.

Cells (at the 60% of confluence) were co-transfected with the S plasmid from SARS-CoV-2 (2 ug/uL), HIV *gag-pol* (1 ug/uL) and the pCSFLW (1.5 ug/uL) using EndoFectin™ Lenti transfection reagent (Tebu Bio—217EF002, Via Pretorio 4—C.P. 70. I-20013 Magenta, Milano) in accordance with the manufacturer’s instructions.

The following day, the supernatants were replaced with DMEM without phenol red (Thermo Fisher Scientific, 168 3rd Ave, Waltham, MA 02451, United States containing 10% FBS, and the plates were incubated for 48 h at 37 °C in an atmosphere of 5% CO_2_. After 48 h, the supernatants of transfected cells were harvested and filtered by Millex-HA 0.45 um filter.

Concurrently, HEK 293T/17 cells were also transfected with VSV-G plasmid (1 μg/μL), and a no-spike control (Δ envelope) was generated by co-transfection with HIV *gag-pol* and pCSFLW plasmids only.

#### 2.3.2. SARS-CoV-2 Pseudotypes Titration

For SARS-CoV-2 titration, a further transfection is required in order to allow pseudotypes to enter target cells.

The day before titration, HEK 293T/17 cells were co-transfected with two plasmids encoding for the ACE2 receptor gene and TMPRSS2, by means of EndoFectin™ Lenti transfection reagent; after overnight incubation at 37 °C, the supernatant was replaced by DMEM containing 10% FBS.

The following day, supernatants were serially two-fold diluted in a fresh cell culture medium in 96-well, flat-bottomed culture plates, and 1 × 10^4^ HEK 293T/17 target cells were added to each well. As controls, VSV-G and Δ-envelope pseudotypes were also included. After 72 h, the luminescence of cell cultures (in Relative Luminescence Units or RLUs) was evaluated by luminometry (Tecan Infinite M1000 Pro Multi-Detection Plate Reader) using the Bright-Glo assay system (Bright-Glo™ Luciferase Assay System, Promega, Viale Piero e Alberto Pirelli, 6, 20126 Milano MI).

### 2.4. p24 Capsid ELISA

Serial dilutions of SARS-CoV-2 pseudotype-containing media were tested by means of the Lenti-X p24 Rapid titre kit (Cat. No. 632200) (Takara, Japan) in accordance with the manufacturer’s instructions.

### 2.5. Western Blotting

In order to verify the incorporation of the S glycoprotein of SARS-CoV-2, the S protein expressed on the lentiviral pseudotypes was detected by Western blot analysis.

Western blot analysis of spike protein was performed on the supernatant from sub-confluent HEK 293T/17 cells co-transfected with HIV *gag-pol* plasmid, CSFLW plasmid and the S plasmid from SARS-CoV-2. Western blot analysis was also performed on LV in a BSL3 facility.

Δ-envelope (no-spike control) pseudotypes prepared with the same procedure were run as a negative control. SARS-CoV-2 pseudotypes, SARS-CoV-2 live virus and Δ-envelope pseudotypes were mixed with SDS sample buffer. The mixture was heated for 10 min at 75 °C and electrophoresis (50 μg of protein/sample) was carried out in 4–12% Bis-Tris Gels (Life Technologies, Carlsbad, CA, USA). Proteins were then blotted onto nitrocellulose membranes and incubated overnight with 500-fold diluted sera from convalescent SARS-CoV-2 patients. A Goat Anti-human IgG (Bethyl, 25043 FM 1097, Montgomery, TX 77356, United States) was used as a secondary antibody (1:5000 dilution). The chemiluminescent signals from the nitrocellulose membranes were captured by a camera system (ImageQuant LAS 400 instrument).

### 2.6. SARS-CoV-2 Pseudotypes Tropism Study

In this study, different cell lines have been tested in order to study their susceptibility to SARS-CoV-2 S protein driven entry, the role of the ACE2 receptor and TMPSSRS2 for S protein priming.

One day before pseudotypes titration, MDCK, Vero E6, Caco2, Hep G2 and Huh7 cells were transfected with the pCAGGS-TMPRSS2 plasmid, while HEK 293T/17 cells were co-transfected with ACE2 and pCAGGS-TMPRSS2 plasmids. After 24 h, the cells were removed by trypsinisation, counted and used for the subsequent titration.

In parallel with SARS-CoV-2 pseudotypes, VSV-G and Δ-envelope pseudotypes were titrated as controls. Plates were incubated for 48–72 h with the pseudotypes, at 37 °C in an atmosphere of 5% CO2, and the efficiency of pseudotypes entry was characterised on the basis of luciferase activity (Relative Luminescence Units or RLUs).

### 2.7. Pseudotype-Based Neutralisation Assays

To measure the neutralisation activity of this panel of sera, neutralising antibody titres were defined as endpoint two-fold seral dilutions of test samples, and the 50% inhibitory concentration (IC_50_) was determined as the serum dilution resulting in a 50% reduction of a single round of infection (reporter gene-mediated signal). Values were expressed as a percentage in comparison with the signal from the cell-only control (equivalent to 100% neutralisation and/or no infection) and the signal from a pseudotype-only control (equivalent to 0% neutralisation or 100% infection).

In brief, two-fold serial dilution of serum samples, starting from 1:10, was performed in a culture medium (DMEM, 5% FBS, 1% PEN-STREP, 1% L-Glutamine). The serum was mixed with SARS-CoV-2 pseudotypes in a 1:1 vol/vol ratio in a 96-well culture plate. The virus input used was 1 × 10^6^ RLU/well (based on the previous titration).

The serum-pseudotypes mixture was then incubated for 1 h at 37 °C in a humidified atmosphere with 5% CO2. After 1 h, HEK 293/ACE2 transfected cell suspensions (1.5 × 10^4^ cell/mL) were seeded into each well of flat-bottomed tissue culture plates. The plates were incubated at 37 °C for 48–72 h, and the neutralising antibodies were characterised on the basis of luciferase activity.

### 2.8. Live Virus, Titration and Microneutralisation Assay

The SARS-CoV-2 strain 2019-nCov/Italy-INMI1-wild-type virus was purchased from the European Virus Archive Goes Global (EVAg, Spallanzani Institute, Via Portuense, 292, 00148–00153, Rome) and propagated in Vero E6 cells. The virus was titrated in a Tissue Culture Infective Dose 50% assay (TCID50) on 96-well culture plates with 1-log serial dilution. The plates were observed daily for a total of four days for the presence of cytopathic effect (CPE). The end-point titre was calculated according to the Spearman–Karber formula [32].

The MN assay was performed as previously reported by Manenti et al. [24]. Briefly, two-fold serial dilutions of serum samples were mixed with an equal volume of viral solution containing 100 TCID50 of SARS-CoV-2. The serum-virus mixture was incubated for 1 h at 37 °C in a humidified atmosphere with 5% CO_2_, then passed to a VERO E6 culture plate. The plates were incubated for four days at 37 °C in a humidified atmosphere with 5% CO_2_. After the incubation time, each well of a 96-well plate was inspected by means of an inverted optical microscope to evaluate the percentage of CPE.

### 2.9. Statistical Analyses

#### 2.9.1. Calculation of SARS-CoV-2 Pseudotype Titres

Pseudotype transduction titres were estimated by means of Excel^TM^ software; the pseudotype titres obtained at each point in a range of dilution points were expressed as RLU/mL, and the arithmetic mean was calculated. For the analyses of pseudotype-based neutralisation assays, titres were firstly normalised, and IC_50_ values were calculated by a non-linear regression model (log (inhibitor) vs. normalised response-variable slope) analysis. Titres were subsequently expressed as the range of dilution in which the IC_50_ value lay. In order to evaluate cell infectivity, two-way analysis of variance (ANOVA) with Dunnett posttest was used to test for statistical significance (*p* > 0.05 (ns, not significant), *p* ≤ 0.05 (*), *p* ≤ 0.01 (**), *p* ≤ 0.001 (***), and *p* ≤ 0.0001 (****)). For all statistical analyses, the GraphPad Prism version 8.4 package was used (GraphPad Software, GraphPad, 2365 Northside Dr., Suite 560, San Diego, CA 92108, USA).

#### 2.9.2. Comparison between Live Virus Microneutralisation Titres (MNT) and Pseudotype Neutralising Titres (PNT)

**Figure 1:**
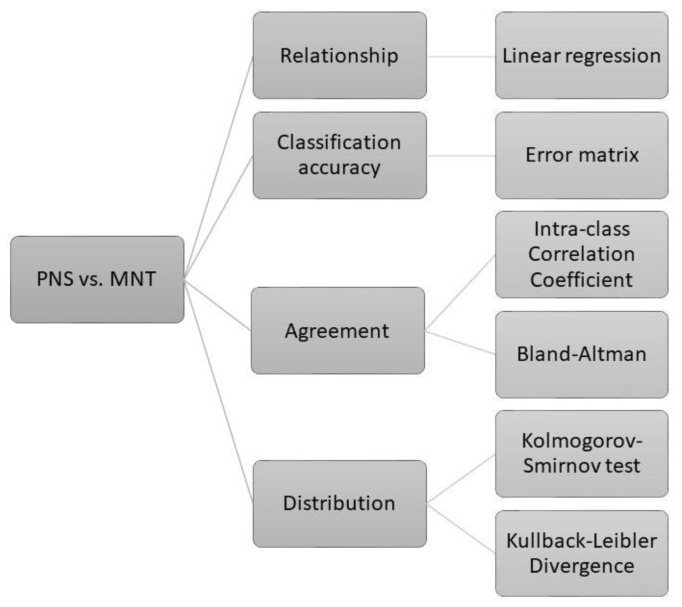
Flowchart of the statistical methods. Comparison between pseudotype neutralising titres (PNT) and live virus micro-neutralisation titres (MNT) was conducted via different approaches, which enabled us to elucidate the overall convergence of the PNT and MNT results.

The statistical analyses have been undertaken with the R software (version 3.6.2). Different approaches drove our statistical analyses, as shown in Figure 1.

All the titres underwent preliminary base-2 logarithmic transformation. In accordance with the classification approach, the titres greater than log2 (5) were labelled as “Positive,” and otherwise as “Negative.” With MNT taken as the reference (“true”) results, the misclassifications were counted and displayed in an error matrix table.

A linear regression model provided a measure of the strength of the relationship between PNT and MNT. As the dependent variable, we considered the log2 of PNT, and as the independent variable, the log2 of MNT.

For the evaluation of the agreement between PNT and MNT, we used the intra-class correlation coefficient (ICC). In addition, the Bland–Altman method (BA) enabled us to search for possible systematic difference (bias) between the PNT and MNT, as well as to identify the presence of outliers. The BA evaluation mainly consists in a scatter plot of the differences between the measurements vs. their means. The 95% limits of agreement (LOAs) were calculated around the mean of the differences. We set a maximum acceptable difference (MAD) of 0.5 times the MNT, below which the observed PNT-MNT differences were considered as not having a significant biological effect. We interpreted the differences below the MAD and within the 95% limits of agreement as interchangeable. By comparing the distributions of PNT and MNT, we got further insight into their similarity degree. Thus, we applied the Kolmogorov–Smirnov test and measured the Kullbac–Leibler divergence (KLD). The former considers the largest difference between the empirical distribution functions of the PNT and MNT tests, and the latter is an information theoretic-based value, which indicates how much information is lost when taking PNT as an approximation of MNT. The KLD was calculated as the “true” reference the MNT distribution, and normalised as follows:

(1) $$nKLD\left(p_{MNT}||p_{PNT}\right)\;=\;\frac{1}{1+e^{KLD}}-\frac{1}{2}$$

where *nKLD* is the normalised divergence, and *pMNT* and *pPNT* are the distributions of *MNT* and *PNT*, respectively. This normalisation restricts the divergence values in the range [−0.5, + 0.5], such that *nKLD* = 0 if the *PNT* distribution perfectly reproduces the *MNT* distribution, while the extremes *nKLD* = −0.5 or *nKLD* = 0.5 are attained if *KLD* tends towards infinite, (positive or negative infinite, respectively). Lastly, we conducted a bootstrap test (100,000 resamples with replacement from the *MNT* and *PNT* data) on the *KLD* statistic under the null hypothesis of *nKLD* = 0.

## 3. Results

### 3.1. SARS-CoV-2 Spike Protein Expression Evaluated by Western Blotting and p24 Quantification

SARS-CoV-2 S glycoprotein and gag-p24 in pseudotypes were characterised by immunoblot analysis and p24 ELISA, respectively.

To verify the expression of the spike protein in the SARS-CoV-2 pseudotypes, the spike was detected by Western blot; sera from convalescent SARS-CoV-2 patients, which have been shown to have a high neutralising titre in microneutralisation with a live virus, were used as the primary antibody, and goat anti-Human IgG as the secondary antibody.

SARS CoV-2 strain 2019-nCov/Italy wild-type virus (LV), which was handled in a level 3 bio-containment facility (BSL 3), was used as positive control in order to evaluate the spike glycoprotein expression, while a Δ-envelope pseudotype, prepared with the same procedure, was used as a negative control.

**Figure 2:**
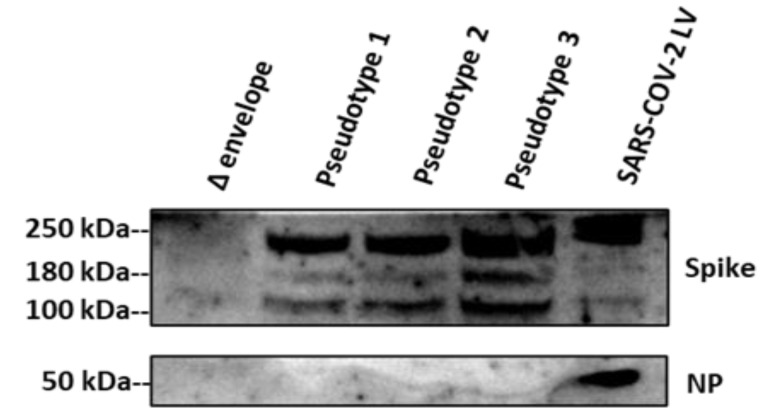
Incorporation of SARS-CoV-2 protein into pseudotypes. The spike protein of the particles was investigated by Western blotting. From left to right: lanes showing Δ-envelope pseudotype; SARS-CoV-2 Pseudotype batch 1, batch 2, batch 3; and SARS-CoV-2 live virus (LV). The LV was used as a positive control and the Δ-envelope (particles bearing no envelope protein), prepared with the same procedure, was used as a negative control. Uncleaved S protein, about 180 kDa; cleaved S protein, about 100 kDa; dimeric-trimeric S protein, above 250 kDa; Nucleocapsid protein, about 50 kDa. Experiments were done twice and one is shown.

Three different batches of pseudotypes were tested; specific bands were found for SARS-CoV-2 pseudotypes and for SARS-CoV-2 live virus, but not for the Δ-envelope control (Figure 2).

Regarding the pseudotypes, we observed three main bands: one just below 250 kDa, and the remaining two bands at 180 kDa and 100 kDa, corresponding to the full-length and cleaved S protein, as shown in previous studies [33].

These two bands (180 kDa and 100 kDa) were barely detectable in the live virus, in which was observed one just above 250 kDa, possibly reflecting the dimeric-trimeric S protein (detected in the pseudotypes below 250 kDa), and the other band was around 50 kDa, possibly corresponding to the Nucleocapsid Protein (NP). The high glycosylation potential to which the spike is subjected during the infection differs from the spike expressed on the pseudotyped particles that do not undergo the same post-translational modifications [34]. This would explain the presence of a protein with a weight greater than 250 kDa in the wild type virus, compared to the three isoforms with detectable molecular weight between 100 KDa and below 250 kDa related to the pseudotypes [33].

Moreover, the quantitative data can slightly differ when a convalescence serum sample is used instead of an antibody (e.g., monoclonal antibody), specifically directed against a defined protein’s portion.

Although, in this study, the evaluation of SARS-CoV-2 pseudotype titres is based on the reporter gene expression (RLUs/mL), a number of eight different batches of SARS-CoV-2 lentiviral pseudotypes were also compared for the HIV-1 viral core protein p24 amount (directly correlating to the number of particles) by ELISA (values reported as pg of p24 for mL).

**Figure 3:**
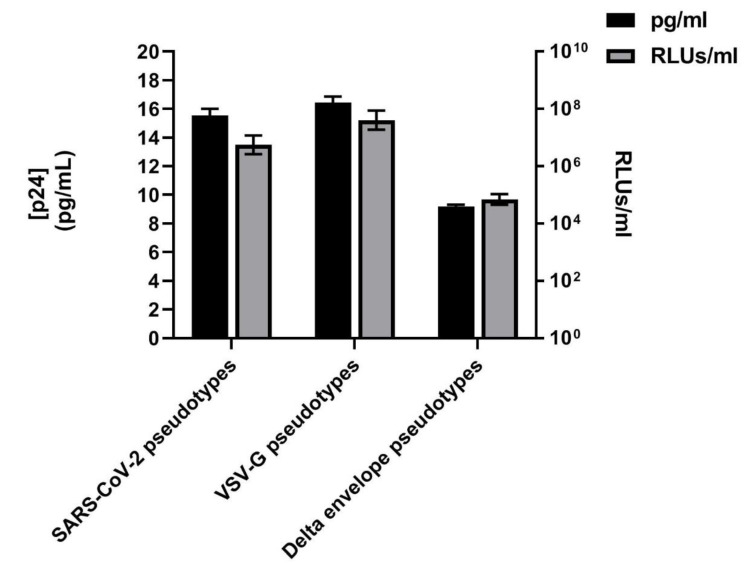
p24 quantification. Vector particle concentration (pg p24 protein per mL) determined by p24 ELISA and corresponding lentiviral vector infectivity (RLUs/mL). Values expressed as the means ± SD of independent measurements (*n* = 8).

The results showed that all batches tested consistently contained around 13–15 pg/mL of p24 gag capsid protein, corresponding approximately to a titre of 1.30e + 05 RLUs/mL. Similar vector infectivity was also identified for VSV-G pseudotyped vectors, around 15–16 pg/mL of p24 gag capsid protein corresponding to an approximate titre of 1.56e + 05 RLUs/mL, while a value of 9–10 pg/mL, corresponding to a titre of 9.94e + 0 4, was obtained for Δ-envelope pseudotypes, as shown in Figure 3. The values obtained for the Delta envelope are slightly higher for the p24 amount compared to the RLUs. A possible explanation, as showed by Geraerts [35], is that p24 quantification by ELISA will detect cores lacking envelope glycoproteins (non-functional) as well as cores belonging to transduction competent (functional) pseudotypes, and this technique usually overestimates the functional vector titre. In fact, it also been shown that omission of the envelope plasmid during the vector production resulted in p24 being comparable with those of a normal production although with a non-detectable functional titre.

### 3.2. Susceptibility of Cell Lines Panel to SARS-CoV-2 for Pseudotype-Based Neutralisation Assays

After the production of the SARS-CoV-2 pseudotypes, we asked which cell lines were susceptible to pseudotype-driven entry in order to have a panel of cell lines that can be used in downstream pseudotype-neutralisation assays. For this purpose, we used a panel of cell lines of human and animal origin.

Since SARS-CoV-2 live virus has been successfully isolated in Vero (African Green monkey kidney cell line), Vero E6 and Huh7 cell lines at high titres (as shown previously [24]), these were chosen for the cell tropism study. The Hep G2, MDCK and Caco cells were also included in this panel because a previous study evidenced ACE2 receptor expression, except for HEK 293T/17 [36]. However, HEK 293/17 cells have also been included as control cell line due to their high transfectability, and they were firstly optimised using different ACE2-expressing plasmid concentrations. Based on these preliminary results, this panel of cell lines was tested against SARS-CoV-2, VSV-G and Delta envelope pseudotypes.

**Figure 4:**
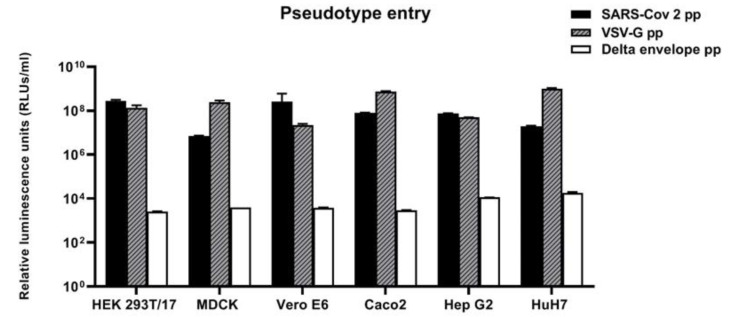
Susceptibility of cell lines to SARS-COV-2 driven entry. Cell lines of human and animal origin infected with Δ-envelope pseudotypes, SARS-COV-2 pseudotypes and VSV-G pseudotypes. At 72 h post-infection, pseudotype entry was analysed by determining luciferase activity in cell lysates. Cell background without pseudotypes infection was used for normalisation. Values are expressed as the means of independent results ± SD (*n* = 3). Two-way analysis of variance (ANOVA) test was used for statistical analysis.

As also seen in previous studies [10,20], all the cell lines tested were highly susceptible to entry driven by VSV-G pseudotypes as demonstrated by titres of 10^7^–10^8^ RLUs/mL (Figure 4). All the cell lines tested were also susceptible to entry by SARS-CoV-2 pseudotypes, in particular, HEK 293 ACE2/TMPRSS2-transfected cells, as demonstrated by titres of 10^8^–10^9^ RLUs/mL. However, no statistical differences have been observed when SARS-CoV-2 pseudotypes have been tested against different cell lines. This comparable susceptibility can be due to the similar ACE2 expression (except for MDCK cell line as also showed by Nie et al. [20]). Moreover, co-transfection with TMPRSS2 protease can potentially level the titres obtained with different cell lines except for HEK 293/17.

As expected, when cell lines were tested with Δ-envelope control (particles without envelope proteins) the transduction activity dropped drastically (4-log), corresponding to 10^3^–10^4^ RLUs/mL (Figure 4).

### 3.3. Correlation between Live Virus MN and Pseudotype-Based Neutralisation Assays

As shown in Table 1, of 65 human serum samples tested, 24 proved positive for PNT, with titres ranging between 10–20 and >1280, and 28 positives for MNT, with titres ranging between 10 and 1280 (Table 2). Forty-one human samples were found negative for PNT and 37 negative for MNT. Therefore, only titres obtained for 4 sera were found to be discordant.

When mABs have been tested against pseudotypes and the live virus, no neutralisation activity was observed on MN, while an evident neutralisation profile was seen for PNT against Human Anti-IgM [37] SARS-CoV-2 spike S1 (IC_50_ > 1280).

In addition, we defined the parameters of sensitivity, specificity and accuracy. From the error matrix (Table 1), we obtained the following results: accuracy = 93.8% (95% CI (85.0%–98.3%)), which was significantly higher (*p* < 0.0001) than the no-information rate (56.9%), sensitivity = 85.7% (95% CI (67.3%–96.0%)) and specificity = 100% (95% CI (90.5%–100%)).

**Figure 5:**
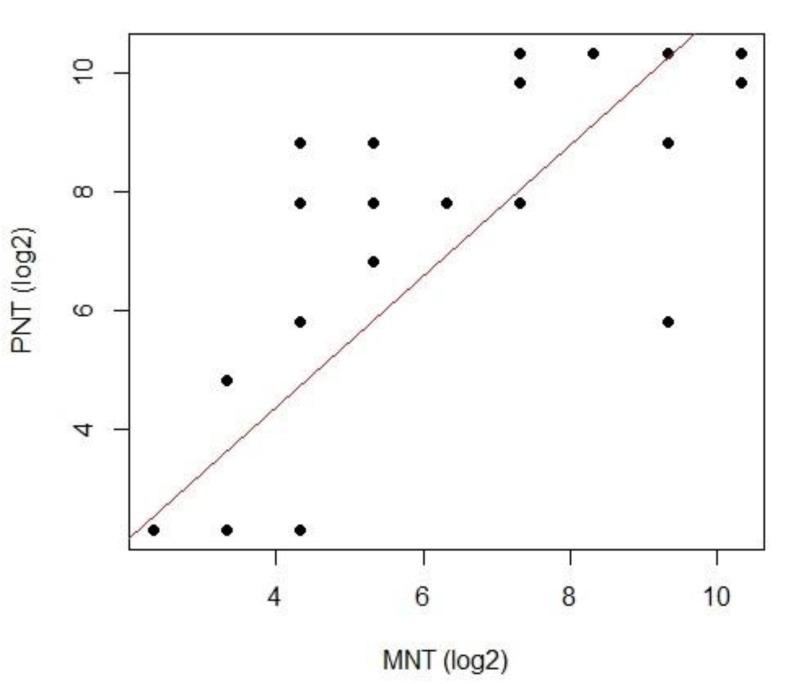
PNT vs. MNT relationship. The scatterplot of the MNT (*x*-axis) and PNT (*y*-axis) presented a linear pattern.

A simple linear regression was conducted to predict the log2 PNT based on the log2 MNT data (Figure 5). The linear regression was found to be significant, F (1, 63) = 344.6, *p* < 0.0001, with an R^2^ = 0.84. The PNT increased 1.09 for each log2 of the MNT.

To evaluate the agreement between PNT and MNT, we used the intra-class correlation coefficient (ICC). The one-way random single score ICC (Table 3) calculated between the neutralisation titres PNT and MNT was 0.872 (95% CI (0.799–0.92)), *p* < 0.0001. This indicated excellent agreement [38].

**Figure 6:**
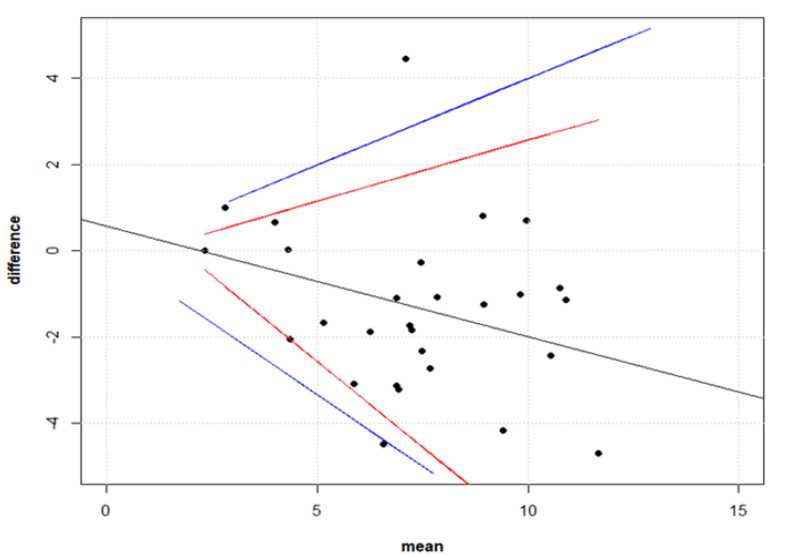
Bland–Altman plot. The Bland–Altman plot evaluating PNT-MNT agreement. The maximum acceptable differences (blue lines) embrace the LOAs (red lines), which makes the interpretation of the statistical results biologically plausible. We found a significant linear relationship between the differences and the means of the titres. Moreover, except for one outlier, all the differences were both biologically and statistically acceptable. In other words, there was substantial agreement between PNT and MNT.

The Bland–Altman (BA) analysis (Figure 6) revealed the presence of a significant relationship between differences and means, which made applying a regression approach consistent [39].

We found that most of the data-points were within the 95% Limit of Agreements (LOAs), while one outlier, corresponding to a means of titres equal to 6.56 (log2-units), was detected. These findings suggested that there was substantial agreement and interchangeability between PNT and MNT.

In addition, the BA method enabled us to search for possible systematic difference (bias) between the PNT and MNT, and to identify the presence of outliers.

**Figure 7:**
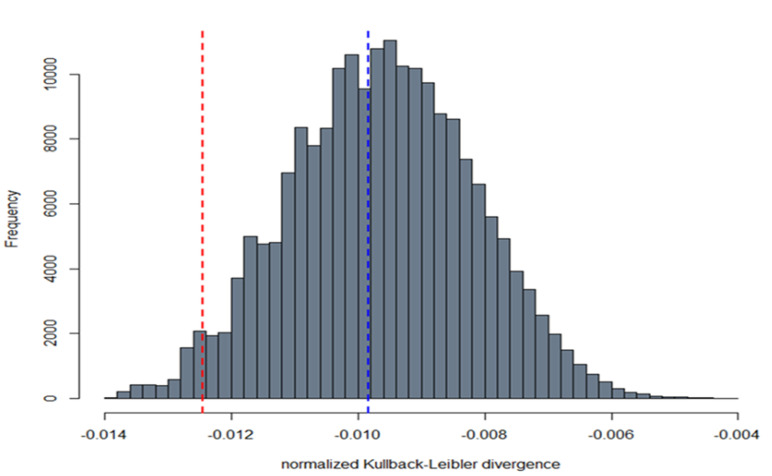
Bootstrap distribution of the Kullback–Leibler divergence. The histogram shows the distribution of the normalised Kullback–Leibler divergences evaluated via the bootstrap method. The nKLD between the original MNT and PNT was −0.0098 (blue dotted line). The MNT and PNT data were re-sampled with replacement 100,000 times. The red dotted line shows the 5% lower-tail quantile (−0.0125) of the bootstrap distribution. Since the observed nKLD was lower (in absolute value) than the 5% lower-tail quantile, we concluded that the divergence was not significantly different from zero (*p*-value = 0.44).

We also concluded, on the basis of the Kolmogorov–Smirnov test, that there was not enough evidence to reject the null hypothesis of equal distributions of PNT and MNT, (KS test = 0.17, *p* = 0.31). The normalised Kullback–Leibler divergences (nKLD) between the original PNT and MNT data was equal to −0.0098. The result of the bootstrap testing evidenced that the hypothesis of a zero nKLD between PNT and MNT was consistent with the data, *p* = 0.44 (Figure 7).

## 4. Discussions

The recent emergence of the novel pathogenic SARS-Coronavirus-2 constitutes a global health emergency. As previously shown [16,21], infection with SARS-CoV-2 elicits antibodies that bind to the virus. Although several studies are still ongoing regarding the virus and the complexity of the human immune responses, neutralising antibodies are known to be strongly correlated with protection [40]. Currently, polyclonal antibodies from recovered SARS-CoV-2-infected patients have been used to treat the SARS-CoV-2 infection, but identification of SARS-CoV-2-specific neutralising mAbs is still ongoing. Once such antibodies are selected and produced, the subsequent steps will involve testing for neutralising and/or cross-neutralising activity [41], which should simplify the analysis of functional humoral immune responses.

The demand for serological testing for SARS-CoV-2 is high, as there is a need to better quantify the number of cases of COVID-19, including asymptomatic carriers [42] and patients who have recovered.

As we know, SARS-CoV-2 have a strong pathogenicity and working with the wild-type virus implies the need for level 3 bio-containment facility, according to the WHO guidelines. However, serological assays for the evaluation of neutralising activity against the SARS-CoV-2 currently require the use of isolated-live virus.

Indeed, high-throughput methods (such as ELISA) that do not require the live virus are limited by the fact that they detect total antibody binding to SARS-CoV-2 or to some of its key constituent proteins [43,44]. For this reason, we produced and characterised a lentiviral pseudotype system that expresses the S protein of SARS-CoV-2 with the same approach used by for other pathogenic coronaviruses including SARS-CoV and MERS [29,30].

This pseudotype system can be a useful tool because of its safety and versatility. Its versatility lies in the fact that the virus can be pseudotyped with different envelope proteins [30,45,46]. Moreover, this approach does not necessitate handling the live virus, and does not require high-level bio-containment facilities, as the pseudotype is devoid of virulent viral components and it is involved in a single round of replication [46].

The production of pseudotypes harbouring novel glycoproteins could permit elucidation of viral biological characteristics and a better understanding if they have potential to cause pandemics by the generation of mutants (via mutation of glycoprotein) without the risk of creating potentially dangerous viruses. It can also reflect key aspects of host cell entry and receptor binding specificity [10,33].

However, the need of additional reagents seems a requirement due to cellular receptor specificity and TMPRSS2 priming for SARS-CoV-2 pseudotypes; thus, necessitating us to optimise the batch-to-batch variations in order to reduce the variability in terms of pseudotype titres and stability.

Since previous studies have evidenced that most amino acid residues for ACE2 binding by SARS-CoV were also conserved in SARS-CoV-2 [47], we have studied different cell susceptibility to SARS-CoV-2 pseudotype entry. Understanding the entry mechanisms determined by the S glycoprotein and the susceptibility of different cells (based on specific cellular receptor expression) can also provide important information to study critical process in which the S spike protein of SARS- CoV-2 is involved. Moreover, the use of multiple target cell lines is particularly valid since one of the advantages of the pseudotype-based assays is that they are deployable across different stakeholder laboratories who have access to different cell lines.

It also represents a determinant for the development and optimisation of cell-based assays and for the screening of potential entry inhibitors.

Once the SARS-CoV-2 pseudotypes have been efficiently produced, we have also developed a lentiviral pseudotype-based assay that facilitates the accurate determination of neutralising antibody responses to SARS-CoV-2 that can be paired to the more intensive and laborious MN.

Although it is unclear as to how the display of S protein on heterologous virus impacts viral entry, antibody recognition and antibody neutralisation [48], our results suggest that pseudotype-based neutralisation assays correlate well with the MN assays when testing human sera, and our findings show significant accuracy, sensitivity and specificity when both assays are compared.

When employed in the screening of vaccinated human sera and other influenza serological studies [49,50,51,52], pseudotype-based neutralisation assays have been described to be more sensitive than classical MN. The sensitivity of pseudotypes can also detect particularly low antibody responses or facilitate the antibody’s recognition that cannot always be determined by conventional assays, possibly due to lower density/quantity of glycoprotein expressed on the pseudotypes [53,54]. This could explain the ability of human mAB IgM to recognise certain epitopes of S1 protein, as it has been seen for pseudotype-based neutralisation but not for live virus MN.

Although the model (PNT instead of MNT) returned a relatively high percentage of false-negatives, in large-scale serological testing, having a highly specific test is advantageous when a false-positive result has a great clinical impact. Indeed, it guarantees against the risk of misclassification of the true-negative samples.

Undoubtedly, for a wider use of pseudotypes, it will be necessary to take into consideration additional aspects required for standardisation such as comparison between viral vectors/expression plasmids used, optimisation of particle titres and establishment of adequate positive, negative controls and reference standards (making it more difficult to evaluate the reproducibility of different serological assays).

Moreover, as in all the cell-based assays, cell input [55,56] is an important aspect in standardisation: the use of an automatic cell counter, cell viability and requirement of different proteases should be taken into account and it could be important for the consistency of the assay during different analytical sessions. Undoubtedly, a permanent cell line could be a valid alternative [18] (it would require only TMPRSS2 priming), and the generation of a cell line expressing proteases as a pseudotype producer could be investigated.

## 5. Conclusions

Our findings suggest that SARS-CoV-2 pseudotype-based assays and the live-virus microneutralisation correlate well when employed in testing antibody responses against the novel SARS-CoV-2 virus. Undoubtedly, they would help to dissect out these diversities of immunological responses, and they can, additionally, have an important role in evaluating the neutralising antibody potency (but not necessarily predicting protection), and in testing the efficacy of potential SARS-CoV-2 vaccines.

## Figures and Tables

**Table 1:** Error matrix. Reliability of PNT compared to MNT in serum samples from 65 subjects. Titres greater than log2(5) were labelled as “Positive”, and otherwise as “Negative”. With MNT taken as the reference (true) results, the table shows the misclassifications.

	MNT		
PNT	Negative	Positive	Total
Negative	37	4	41
Positive	0	24	24
Total	37	28	65

**Table 2:** Panel of human sera tested by live virus MN and pseudotype-based neutralisation assays. Responses against SARS-CoV-2 are expressed as antibody titres for both assays (end-point dilution).

ID Samples	Live Virus Micro	Pseudotype Neutralisation
	Neutralisation Titres (MNT)	Titres (PNT)
1	5	<10
2	5	<10
3	5	<10
4	5	<10
5	5	<10
6	5	<10
7	5	<10
8	5	<10
9	5	<10
10	5	<10
11	10	10–20
12	10	<10
13	10	<10
14	20	40–80
15	20	40–80
16	20	<10
17	20	<10
18	20	160–320
19	40	160–320
20	40	160–320
21	40	80–160
22	80	160–320
23	80	160–320
24	80	320–640
25	80	160–320
26	80	160–320
27	160	160–320
28	160	160–320
29	160	>1280
30	320	640–1280
31	640	>1280
32	640	20–40
33	640	320–640
34	640	>1280
35	640	>1280
36	1280	>1280
37	1280	>1280
38	1280	640–1280
39	5	<10
40	5	<10
41	5	<10
42	5	<10
43	5	<10
44	5	<10
45	5	<10
46	5	<10
47	5	<10
48	5	<10
49	5	<10
50	5	<10
51	5	<10
52	5	<10
53	5	<10
54	5	<10
55	5	<10
56	5	<10
57	5	<10
58	5	<10
59	5	<10
60	5	<10
61	5	<10
62	5	<10
63	5	<10
64	5	<10
65	5	<10

**Table 3:** Summary of the intra-class correlation analysis. The one-way random single score ICC calculated between the neutralisation titres PNT and MNT was 0.872 (95% CI (0.799–0.92)), *p* < 0.0001.

Model	one-way
Type	agreement
Subjects	65
Raters	2
ICC(1)	0.872
CI 95%	[0.799–0.92]
*F*-test	14.7
*p*	<0.0001
